# Troubling Toys: Rare-Earth Magnet Ingestion in Children Causing Bowel Perforations

**DOI:** 10.1155/2014/908730

**Published:** 2014-04-30

**Authors:** Parkash Mandhan, Muthana Alsalihi, Saleem Mammoo, Mansour J. Ali

**Affiliations:** Department of Pediatric Surgery, Hamad General Hospital, Hamad Medical Corporation, P.O. Box 3050 Doha, Qatar

## Abstract

Ingestion of foreign bodies in the pediatric population is common and magnet ingestion is known to cause a significant morbidity. Rare-earth magnets are small 3–6 mm diameter spherical powerful magnets that are sold as popular desk toys for adults and were previously found in construction toys in attractive colors for children to play with. We describe 2 young healthy children who ingested rare-earth magnets Buckyballs while playing with these magnetic toys and later presented in emergency with acute abdomen. Abdominal imaging revealed several (26 and 5) pieces of rare-earth magnets in the bowel loops. Emergency surgical exploration revealed multiple gastrointestinal perforations and fistula formation at sites of bowel entrapment in between strong magnets apposed to one another. We highlight the potential dangers of rare-earth magnets in children and suggest increasing public awareness about risks involved in rare-earth magnets ingestion by children to overcome this serious public health issue.

## 1. Introduction

Ingestion of various types of foreign bodies such as coins, toy parts, jewelry pieces, needles and pins, fish and chicken bones, and button-type batteries is common among children [1]. Reports of magnet ingestion are increasing rapidly globally and over 150 cases have been reported over 22 countries in 2012 [2–4]. Rare-earth magnets, also known as Buckyballs (Maxfield and Oberton, New York, NY), are small 3–6 mm diameter spherical powerful magnets that are sold as popular “desk toys” for adults and were previously found in construction toys in attractive colors for children to play with. Although the US Consumer Product Safety Commission, the American Academy of Pediatrics, and the North American Society for Pediatric Gastroenterology have highlighted growing concerns over these high-powered magnets [4, 5], the current popularity of these magnets as toys and also as body ornamentation for tongue and lip piercing has heightened the safety concern of these magnets in all pediatric age groups resulting in a serious public health issue.

We describe 2 young healthy children who inadvertently ingested multiple pieces of rare-earth magnets while playing with these magnets and developed serious complication requiring emergency surgical intervention. We recommend more public awareness and information to parents and physicians about the potential risks of these magnetic toys.

## 2. Case 1

**Figure 1:**
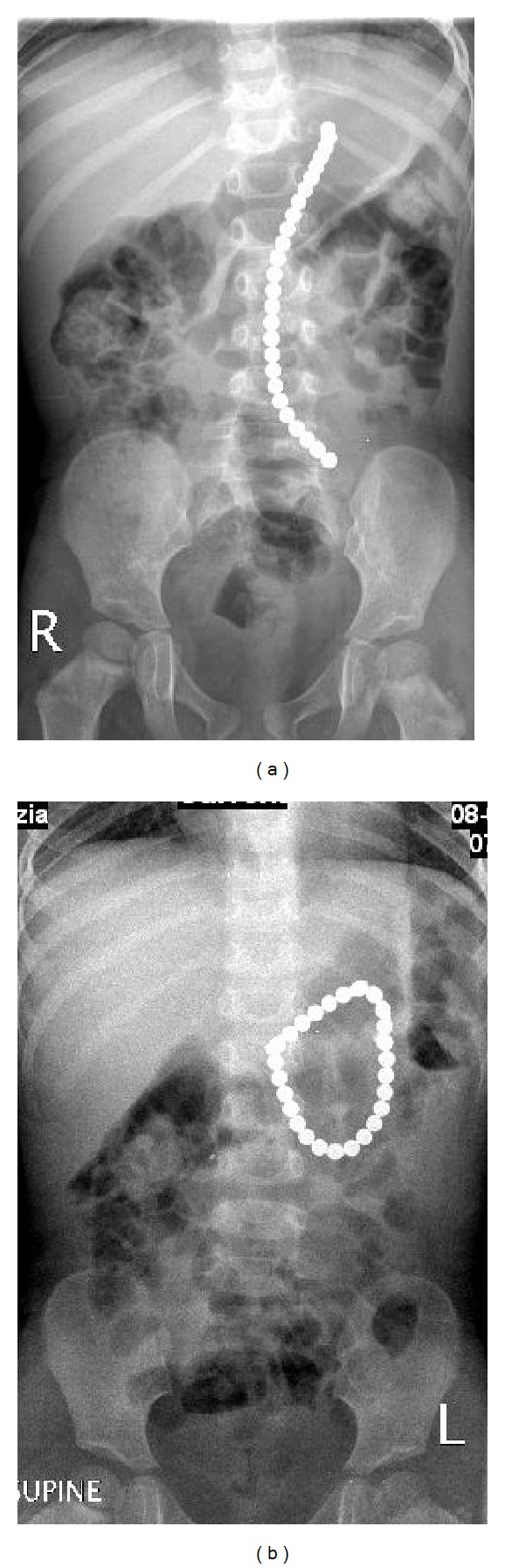
(a) Plain X-ray of abdomen of 2-year-old girl showing ingested 26 pieces of rare-earth magnets joined together in linear fashion. (b) Repeated plain abdominal X-ray of the same patient after 36 hours showing ingested rare-earth magnets forming a ring in the left upper abdomen with no free air and/or obstructive bowel pattern.

**Figure 2:**
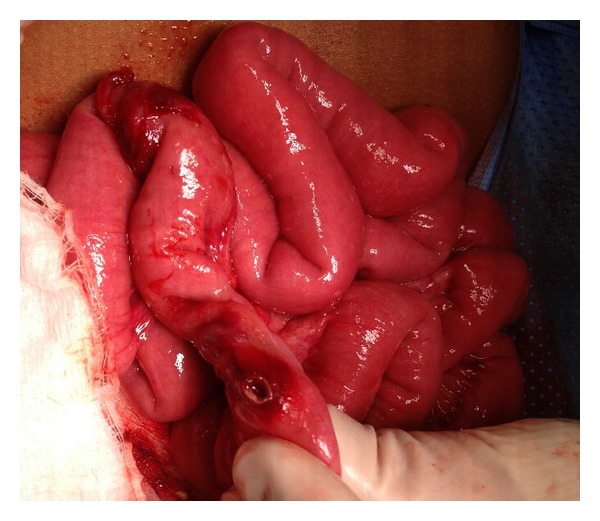
Operative findings of the same patient showing loops of jejunum, which were entrapped in between multiple rare-earth magnets resulting in pressure necrosis and bowel perforation.

A 2-year-old girl was admitted through accident and emergency room with a short history of abdominal pain. The initial clinical and laboratory assessment of patient was unremarkable and a plain X-ray of abdomen showed 26 pieces of rare-earth magnets joined to each other in linear fashion in the left upper quadrant (Figure 1(a)). Further exploration from parents revealed that prior to this she was playing with a box of rare-earth magnets with her 5-year-old brother. The patient was kept under close observation and a repeat abdominal X-ray after 6 hours showed that all pieces of rare-earth magnets still joined together and are present in the left upper part of abdomen. After 12 hours, the patient developed vomiting and showed tachycardia, mild dehydration, and guarding in the midabdomen. Another X-ray of abdomen showed 26 magnetic pieces forming a ring in the left upper abdomen with no pneumoperitoneum and/or obstructive bowel pattern (Figure 1(b)). After discussion with parents, the child was taken to operating room for laparoscopy and proceeds to remove the magnets. Laparoscopy showed multiple small bowel loops adherent to each other forming a mass in the left upper quadrant. The procedure was converted to open through umbilical port site. On careful examination, it was observed that loops of jejunum are entrapped in between multiple magnetic pieces inside the jejunum resulting in pressure necrosis and perforation of jejunum at two sites (Figure 2). Through this enterotomy site, 14 pieces of magnets were retrieved and the remaining 12 pieces were not palpable in the small and large bowel. A table X-ray showed these missing pieces of magnets in the stomach, which were palpated and retrieved through a gastrostomy. Her postoperative course was unremarkable and she was reviewed in clinic after 2 weeks. Six months after initial surgery, the patient was brought back to accident and emergency room with symptoms of bowel obstruction, which was confirmed by the radiology images. After adequate resuscitation and a period of observation, the patient was taken to operating room for emergency reexploration of abdomen, which showed multiple adhesions resulting in bowel obstruction requiring adhesiolysis. The patient's postoperative recovery was slow and was discharged after full recovery. She has been reviewed in our follow-up clinic and has remained stable.

## 3. Case 2

**Figure 3:**
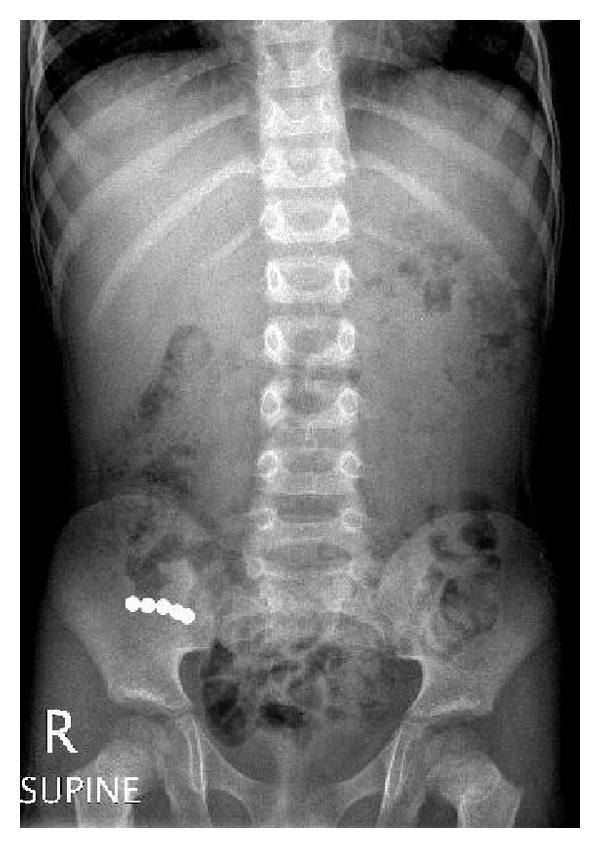
Plain X-ray of abdomen of 4-year-old boy showing ingested 5 pieces of rare-earth magnets joined together due to strong magnetic force.

A 4-year-old boy had mild occasional abdominal pain 3 days after ingestion of rare-earth magnets while playing. He was brought to accident and emergency room and his initial examination was unremarkable. A plain X-ray of abdomen showed 5 pieces of rare-earth magnets in the right lower quadrant joined together (Figure 3). The patient was admitted and parents were informed about the risks related to rare-earth magnet ingestion and offered early surgical intervention to retrieve the magnets but the family refused and opted to observe the spontaneous passage. After 24 hours of observation, the parents were counseled again for potential risks and after that the child was taken to operating room where initial laparoscopy revealed adhesive loops of small bowel and a perforation in the distal ileum. The procedure was converted to open through supraumbilical port site and 5 pieces of rare-earth magnets were retrieved through the perforated ileum. Patient's postoperative course was uneventful and he has been reviewed regularly in the follow-up clinic.

## 4. Discussion

Multiple magnet ingestion in young children poses a serious health risk [3, 6]. When ingested inadvertently, a single piece of rare-earth magnet is expected to behave like other foreign objects and often observed to pass spontaneously, whereas ingestion of multiple magnet pieces is known to cause potential complications [4]. New generation of powerful rare-earth magnets consists of alloys of neodymium iron boron or samarium cobalt, which results in strong magnetic force. These magnets are capable of attracting each other through up to 6 layers of bowel wall and are strong enough to reposition the intestines in order to meet [7]. In one reported case, a 2-year old died of sepsis before rare-earth magnet ingestion was discovered and treated [8]. In our study, both children developed bowel perforations in a short period of time after ingestion of magnets possibly due to pressure necrosis of trapped bowel in between forceful magnetic attraction of rare-earth magnets. Both children required emergency surgery to remove multiple pieces of magnets. Even though the recovery from initial surgery was smooth in both cases, one patient represented within six months with bowel obstruction requiring second surgery. This highlights that the early intervention to remove multiple pieces of such powerful magnets may not reduce the high risk of complication related to ingestion of rare-earth magnets.

The number of magnets ingested by children has been described from 2 to 5 and only in few cases the incident of ingestion has been reported to be witnessed [6, 9–11]. In our study, one child ingested 26 and other 5 pieces of magnets and parents of both children were not aware of the ingestion. It is anticipated that, due to young age and lack of witness, the time interval between the ingestion and the date of intervention varied, which may have contributed to the perforation and fistula formation. Other contributory factors include the nature of magnets as recently engineered magnets contain iron, boron, and neodymium powders that are five to 10 times stronger than plain iron magnets [12]. Therefore, the bowel walls that are compressed between these strong magnets nearly disappear resulting in fistula formation, leakage, and peritonitis.

In the past, children with a variety of psychological conditions such as autism, developmental delays, history of pica, schizoid characteristics, behavioral problems, mental retardation, reactive attachment, and anxiety were considered to be at high risk for accidental ingestion of such objects [9]. At present, the incidence of this problem no longer remains confined to these children as both of our patients were well developed with no psychosocial condition. The possible causes for the increase of ingestion of such magnets include easy availability, attractive colors, small size, and making of these magnets along with poor visible risk information on boxes/toys and lack of national warnings. In our patients, the younger patient ingested multiple pieces of rare-earth magnets while her elder sibling was playing with her and the second child owned these magnets and ingested them while playing. A visible and strong readable warning over the package along with release of periodic information about such cases from national and regional consumer product safety authorities in local print and electronic media about the risks for children and adolescents involved in the use of toys/material with high-powered rare-earth magnets will enhance the awareness about this serious public health issue and will significantly contribute to the prevention of such cases.

## 5. Conclusion

Ingestion of multiple rare-earth magnets leads to serious gastrointestinal morbidity even with early intervention. Concerns regarding magnet ingestion in young children have increased because of the current popularity of new generation high-powered rare-earth magnets, and our two cases highlight the potential hazards and associated gastrointestinal complications of ingestion of such magnets in the young children.
